# ELIXIR pilot action: Marine metagenomics – towards a domain specific set of sustainable services

**DOI:** 10.12688/f1000research.10443.1

**Published:** 2017-01-23

**Authors:** Espen Mikal Robertsen, Hubert Denise, Alex Mitchell, Robert D. Finn, Lars Ailo Bongo, Nils Peder Willassen

**Affiliations:** 1 Center for Bioinformatics (SfB), UiT The Arctic University of Norway Bioinformatics, Tromsø, Norway; 2 European Molecular Biology Laboratory, European Bioinformatics Institute (EMBL-EBI), Wellcome Genome Campus, Cambridge, UK

**Keywords:** Marine, metagenomics, pipelines, gap analysis

## Abstract

Metagenomics, the study of genetic material recovered directly from environmental samples, has the potential to provide insight into the structure and function of heterogeneous microbial communities.  There has been an increased use of metagenomics to discover and understand the diverse biosynthetic capacities of marine microbes, thereby allowing them to be exploited for industrial, food, and health care products. This ELIXIR pilot action was motivated by the need to establish dedicated data resources and harmonized metagenomics pipelines for the marine domain, in order to enhance the exploration and exploitation of marine genetic resources. In this paper, we summarize some of the results from the ELIXIR pilot action “Marine metagenomics – towards user centric services”.

## Introduction

Marine microbial genomics and metagenomics are arguably still in their infancies, but each discipline is rapidly expanding in terms of research activity and are converging against each other. At present, the lack of specialized databases for marine metagenomics ^1^, as well as dedicated data management e-infrastructures and harmonized pipelines, makes implementation of large-scale studies challenging, and replication of analysis close to impossible. In addition, data production from metagenomics projects is growing exponentially due to reducing sequencing costs ^2^, which demands optimized and flexible solutions for analysis of metagenomic data.

To address these challenges, UiT The Arctic University of Norway (part of ELIXIR Norway), together with the European Molecular Biology Laboratory, European Bioinformatics Institute (EMBL-EBI), initiated an ELIXIR pilot action with the following aims: (i) Identify the overlap between two existing metagenomics pipelines, EBI Metagenomics Portal (EMG) ^3^ and META-pipe ^4^, thereby opening the potential for interoperability; (ii) implement new or improve existing components in each pipeline to enrich the output; and (iii) perform a gap analysis to identify deficient areas of marine metagenomics analysis. The overall outcome of this pilot action was aimed at shaping the foundations from which the marine (meta)genomics community could establish long-term, sustainable service platforms. A description of this pilot action and a webinar video can be found at https://www.elixir-europe.org/about/implementation-studies/marine-metagenomics and https://www.elixir-europe.org/documents/update-elixir-pilot-actions-launched-2014-marine-metagenomics-towards-user-centric, respectively.

The EMBL-EBI has developed EMG, a generic platform, which aims to provide insights into the phylogenetic diversity and functional potential of all environmental samples, while UiT has specifically developed META-pipe towards the marine domain, with a focus on bioprospecting. In this article, we describe a comparison of the two pipelines, using the outputs of equivalent input sequence data to illustrate the similarities and differences.

### Overview of pipeline workflows

**Figure 1.**
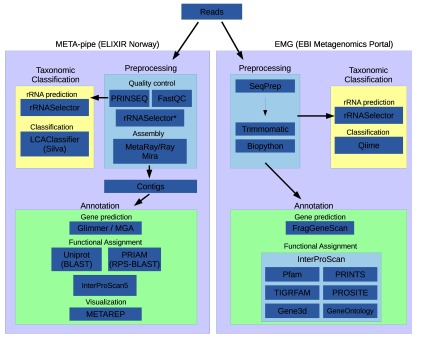
Tools and steps in EMG and META-pipe.

Figure 1 shows a schematic of the pipeline workflows from META-pipe and EMG. A simple visual comparison of these workflows reveals that while there are some commonalities between the pipelines, there are a series of key differences in the tools and approaches to the analysis.

Briefly, the main differences between the pipelines are in the preprocessing and taxonomic classification steps. More specifically, while both preprocessing steps preform filtering of low quality reads and length filtering they diverge thereafter. META-pipe performs assembly of small-subunit (SSU) ribosomal RNAs (rRNAs) filtered reads, whereas EMG merges overlapping pair-end reads into longer single reads (where appropriate) and performs taxonomic classification and functional analysis on unassembled sequences. Despite the presence or absence of sequence assembly, both pipelines use rRNASelector ^5^ for the identification of 5S, 16S and 23S rRNA sequences, before passing extracted rRNAs to taxonomic classification tools. EMG uses QIIME ^6^ with Greengenes ^7^ and a closed-reference OUT picking strategy for taxonomic classification and annotation of 16S rRNA, while META-pipe uses LCAClassifier ^8^ coupled with a manually curated custom database coined SilvaMod, derived from SILVA ^9^ and especially created for LCAClassifier. EMG masks all rRNA regions before passing them to the functional analysis section of the pipeline, whereas META-pipe removes all rRNA prior to assembly. There are also some minor differences between the pipelines in functional annotation. EMG uses FragGeneScan ^10^ for gene prediction and a subset of the InterPro database together with the InterProScan5 ^11^ for functional assignment of predicted coding sequences (CDSs). META-pipe use MetaGeneAnnotator (MGA) ^12^ for gene prediction and InterProScan5 with the full InterPro database, and BLAST against PRIAM ^13^ and UniProt ^14^ for additional functional assignment.

To understand the impact of the outlined differences in functional and taxonomic identifications, and as a prelude to future harmonisation, we undertook a comparison of the results from four different environmental datasets using EMG v2.0 and META-pipe. In addition, as a part of the ELIXIR pilot action project, we performed a gap analysis and concluded on recommendations for developing sustainable ELIXIR services for marine metagenomics domain.

## Methods

### Metagenomics datasets used in the study

For comparison of the two pipelines, four previously unpublished environmental datasets were selected and run against both pipelines. These include two environmental samples from sediments at two different locations in the Barents Sea. Two samples, "Muddy" from the southeast of Edgøya (N77 08 40, E26 31 16) and "Sandy" from the intertidal zone at Nordenskioeldøya located in the Hinlopen Strait (N79 12 49, E19 18 58), were collected during two research cruises in 2010 and 2012, respectively. Sequencing libraries were constructed using the Nextera XT DNA Library Preparation Kit and the Nextera XT Index Kit (Illumina Inc). The samples from the Barents Sea was sequenced with the Illumina MiSeq platform using the MiSeq Reagent Kit v2 (500 cycle) with 2 × 250 bp paired-end read length configuration. The other two samples were from moose and sea urchin; frozen moose faeces found in Grunnfjorden (N69 59 42, E19 36 16) and faeces from a seawater tank containing sea urchin at Nofima (Tromsø), respectively. These two latter samples were sequenced using MiSeq Reagent Kit v3 (600 cycle) in a 2 × 300 bp paired-end read length configuration ( Supplementary Table 1). The metagenomic sequence reads have been deposited at the European Nucleotide Archive ( http://www.ebi.ac.uk/ena) under the sample accession numbers ERS624612 (muddy), ERS624613 (sandy), ERS624611 (moose) and ERS738393 (sea urchin).

### Ribosomal RNA filtering and assembly

Filtering of homologous sequences may reduce the assembly complexity and the number of misassembled contigs. Since META-pipe, contrary to EMG, uses assembled reads (contigs) for functional analysis, we wanted to investigate the effect of removing small-subunit (SSU) ribosomal RNAs (rRNA) sequences before assembly.

To filter prokaryotic rRNA reads, including 5S, 16S and 23S rRNA, hidden Markov models (HMMs) from the rRNASelector were implemented as a part of the functional annotation pipeline. HMMs identify metagenomic fragments coding for rRNA genes if they meet the following two conditions: (i) a sequence read shows an overlap (>60 bp) with an rRNA HMM profile and (ii) the E-value is below 10 ^-5^. Fragments satisfying these conditions were selected. The unselected fragments are stored for subsequent assembly and functional analyses.

All datasets, with or without rRNA filtering, were assembled using MIRA (version 4.0.2) in *de novo* mode, with kmer 31 and forced non-IUPAC bases ^15^.

Before filtering and assembly, the datasets were quality checked with FastQC (version 0.11.3; available at http://www.bioinformatics.babraham.ac.uk/projects/fastqc/) and filtered with PRINSEQ (version 0.20.4) ^16^ (parameters: –trim_left 10 –trim_right 10 –min_len 50 –ns_max_p 10) for all datasets. Additionally, datasets with particularly low quality at the 3’ end (under Q20) were trimmed using parameters –trim_qual_right 20.

### Evaluation of metagenome assemblies

For evaluation of the rRNA filtering step, the assemblies with or without filtering, MetaQUAST v3.2 was used ^17^. For the two sediment samples, MetaQUAST was run in the reference-based evaluation mode with an in-house generated marine reference database, MarRef, as reference genomes. MarRef consists of 337 manually curated complete prokaryotic genomes (unpublished, curated by Terje Klemetsen), with a total length of 1135 Mb ( Supplementary Table 2). For the two faecal metagenomes, moose and sea urchin, MetaQUAST was run in *de novo* evaluation mode. In this case, instead of using a reference database, MetaQUAST downloads reference sequences automatically based on rRNA sequence alignments. To do so, MetaQUAST searches the SILVA rRNA database using BLASTN with contigs as queries, thereby identifying species present in the dataset. The genomes of these species are then downloaded from NCBI and used as a reference database for assembly evaluation. For these latter samples, MetaQUAST identified 64 reference genome sequences with a total length of 262.8 Mb ( Supplementary Table 3).

### Taxonomic classification

The four datasets selected for comparison were run on both pipelines with default parameters. The “Muddy” and the “Moose” dataset were analysed in depth, as we wanted to examine any particular differences using the two pipelines with respect to both marine and gut biomes. EMG and META-pipe both use rRNASelector for selecting rRNA sequences from metagenomics shotgun reads. META-pipe uses LCAClassifier with default parameters (LCA relative range: 2%; minimum bit score: 155) for rRNA annotation, which uses the manually curated Silva *Mod* database – a database based on the taxonomical annotation used in SILVA ^9^ SSURef NR release 106. The Silva *Mod* also includes annotations to the NCBI taxonomy database to increase resolution of eukaryotic classifications based on mitochondrial and plastid 16S rRNA sequences. It offers resolution down to genus rank and has been shown to perform especially well on environmental datasets ^8^. EMG uses QIIME for taxonomic classification, with GreenGenes ^7^ version 13.8 database as a reference for the classification (default closed-reference OTU picking protocol with reverse strand matching enabled). Unique taxa identified were counted for each analyses and results was visualized using Krona charts ^18^.

### Annotation: Gene prediction and functional assignment

META-pipe uses MetaGeneAnnotator (MGA) for prediction of protein-coding (CDS) regions in contigs longer than 500 bp after assembly with MIRA. The MGA uses a self-training model from input sequences for predictions, in addition to statistical models of bacterial, archaeal and prophage genes. The MGA not only sensitively detects typical genes, but also detects atypical genes, such as horizontally transferred genes and prophage genes in prokaryotic sequences. EMG uses FragGeneScan, which combines sequencing error models and codon usages in a hidden Markov model for the prediction of protein-coding region, regardless of species.

For functional assignment of predicted CDSs, EMG uses a sub set of InterPro release 50.0 (Pfam ^19, 20^, TIGRFAM ^21^, PRINTS ^22, 23^, PROSITE patterns ^24^, CATH-Gene3d ^25^), while META-pipe uses the full InterPro release 5.10-50.0, in addition to BLAST against PRIAM version 2.0 and UniProtKB release 2014_09 databases. Gene ontology (GO) terms for all predicted CDSs in the “Muddy” dataset obtained from InterProScan5 were converted to GO-slim terms using OBO-files maintained by the Gene Ontology Consortium ^26, 27^, and used for functional comparison between META-pipe and EMG. This dataset was selected for in-depth functional comparison to emphasize the marine topic of this pilot project.

## Results and discussion

### Assembly quality assessment of rRNA filtering

Assembly of metagenomics reads is a complex and challenging task, due to both the computational overheads and biological complexity. Near-identical sequences, such as mobile genetic elements, homologous genes and conserved regions, combined with high diversity, low coverage and short reads, often results in errors and chimeric assembly. To analyse the effect of filtering rRNA in the assembly process in META-pipe, we assembled four datasets with and without rRNA reads.

Using MetaQUAST to evaluate the effect of rRNA filtering ( Table 1), we observed a marginal reduction in total number of contigs and total length on the rRNA filtered datasets compared to unfiltered. Both marine sediment rRNA unfiltered datasets contain more possible misassembled contigs compared to the corresponding rRNA filtered dataset, 4 and 8 for Muddy and Sandy datasets, respectively. While the Muddy sample contains no misassemblies, the Sandy dataset contains four misassembles, where flanking sequences may align to different reference genomes, overlap or aligns over 1kb away from each other. Similarly, the unfiltered rRNA datasets have more mismatches and indels compared to the two filtered rRNA assemblies. For the Muddy sample, a reduction of mismatches and indels by a factor of 4 was observed for the filtered dataset, while the Sandy gave a reduction of a factor of 3. We believe these mismatches and indels stem from the inherent conservation of rRNA sequences, which causes spurious contigs in assembly.

**Table 1.** Effect on assembly with rRNA filtering.

	Muddy ^1^	Muddy ΔrRNA ^1^	Sandy ^1^	Sandy ΔrRNA ^1^	Moose ^1^	Moose ΔrRNA ^1^	Sea Urchin ^1^	Sea Urchin ΔrRNA ^1^
**# Contigs > 0 bp**	267 433	266 814	148 228	147 928	973 097	972 462	1 010 649	1 010 610
**# Contigs > 500 bp**	25 581	25 302	25 294	25 138	211 333	210 348	114 307	114 189
**# Contigs > 1000 bp**	5 659	5 572	6 213	6 118	57 779	57 147	32 593	32 433
**Total length (bp)**	25 155 475	24 822 906	25 301 011	25 038 101	248 491 504	246 880 376	143 551 468	143 216 805
**Aligned to reference** **(bp)**	291 296 (0.003%)	181 694 (0.001%)	444 398 (0.009%)	286 608 (0.004%)	4 266 146 (0.099%)	3 504 213 (0.083%)	11 923 718 (0.284%)	11 615 439 (0.262%)
**N50 (contigs>500)**	931	930	976	972	1214	1211	1287	1285
**# Misassemblies**	0	0	4	1	10	4	38	20
**# Possible** **misassembled** **contigs**	4	2	8	1	23	5	89	68
**# Mismatches**	918	214	2 836	1 093	5 609	4 202	15 674	14 132
**# Indels**	83	19	277	96	278	167	1003	814

^1^MarRef database length: 1 135 Mb, ^2^MetaQUAST downloaded reference database length: 262 Mb.

A very low percentage of the assembled contigs from the marine sediment samples mapped to the reference genomes (0.001% – 0.009%). However, as MarRef is still relatively small compared to the huge diversity estimated in marine sediments, the low percentage of mapped contigs is not surprising. Consequently, it is difficult to achieve a thorough estimate of misassemblies, mismatches and indels simply because of poor reference coverage. We believe that the number of misassemblies will increase as the marine reference database increases. The marine sediment datasets were also tested using MetaQUAST in *de novo* evaluation mode, where references are identified and downloaded automatically. MetaQUAST generated a reference database of 40 genomes, but assembled contigs only mapped to one of these identified references (in comparison to 159 out of 337 using the in-house marine reference database).

For the faecal datasets, contigs are longer, and more contigs mapped to the reference database (0.083% – 0.286%), which probably is a consequence of higher coverage. However, the number of misassemblies, possible misassemblies, mismatches and indels increased significantly, although the MetaQUAST generated reference database for these samples were considerably smaller than MarRef.

Removal of rRNA before assembly clearly reduces misassemblies, possible misassembled contigs, mismatches and indels, but the lack of specific marine databases hampers the comparison and benchmarking of the different approaches using MetaQUAST.

### Taxonomic classification

In general, META-pipe with the LCAClassifier/Silva *Mod* configuration identifies more unique taxa for the marine sediment datasets, while EMG, using Qiime/GreenGenes, identifies more taxa for the faecal datasets, as shown in Table 2. As LCAClassifier generally offers resolution up to genus rank, we also observe that META-pipe is more reluctant to classify at species level, compared to EMG.

**Table 2.** Number of identified unique taxa. Numbers in parenthesis includes eukaryotic hits classified by META-pipe.

Dataset	Muddy		Sandy		Moose		Sea urchin	
Pipeline	META-pipe	EMG	META-pipe	EMG	META-pipe	EMG	META-pipe	EMG
**Phylum**	41 (57)	40	22 (38)	16	16 (26)	17	18 (54)	13
**Class**	67 (88)	83	38 (62)	36	22 (39)	33	38 (97)	30
**Order**	126 (150)	111	69 (98)	61	32 (51)	42	72 (135)	68
**Family**	113 (138)	97	93 (115)	84	44 (62)	61	88 (157)	107
**Genus**	111 (129)	79	138 (155)	120	79 (92)	85	160 (227)	170
**Species**	6 (11)	19	31 (40)	38	5 (14)	30	61 (102)	73

Our results are in agreement with Lanzen *et al.* ^8^, who showed that classification using Silva *Mod* performed better than with GreenGenes, particularly when applied to environmental sequences. META-pipe also offers eukaryotic classifications based on mitochondrial and plastid 16S rRNA sequences. However, in general, SSU rRNA gives limited resolution as a taxonomic marker for eukaryotic sequences compared to internal transcribed spacers (ITS) or large subunit (LSU) rRNA ^28^.

**Figure 2.**
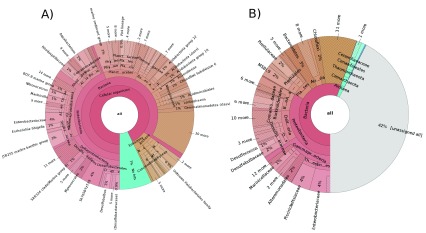
Krona chart representation of taxonomic classification of the “Muddy” dataset from META-pipe ( **A**) and EBI Metagenomics Portal ( **B**) pipelines.ppl.

**Figure 3.**
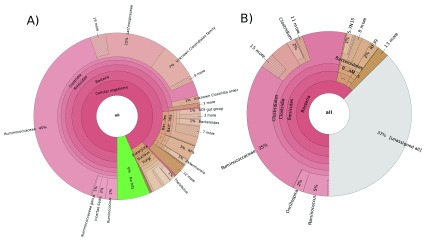
Krona chart representation of taxonomic classification of the “Moose” dataset from META-pipe ( **A**) and EBI Metagenomics Portal ( **B**) pipelines.

To obtain a more detailed overview of the difference between the pipelines, we explored the marine "Muddy" dataset and the gut/intestine “Moose” dataset in more depth. While META-pipe was able to predict 6584 16S rRNA sequences, EMG predicted 4339 in the “Muddy” dataset ( Figure 2). For the “Moose” dataset, META-pipe predicted 43949 and EMG predicted 25018 ( Figure 3). As this step is in practice identical for both pipelines, the dissimilarities in rRNA prediction stems from the preprocessing step in EMG, where overlapping reads are merged and the total read count reduced from 18 to 12 million. Reduction of input sequence reads by one third also reduces predicted rRNA sequences by the same fraction. Although there were dissimilarities in the number of predicted 16S rRNA, the most apparent difference observed between the pipelines was the fraction of unassigned sequences.

In the “Muddy” dataset, EMG classified 2500 sequences (58%), while META-pipe was able to classify 6119 (93%). However, if we ignore unassigned and eukaryotic sequences from the data from META-pipe, most high-level nodes in the taxonomy hierarchy have comparable relative fractions, e.g. like *Planctomycetes*, *Bacteroidetes*, *Acidobacteria*, *Choloflexi*, *Nitospirae* and *Actinobacteria*. The largest inconsistencies were in the *Archaea* and *Protobacteria* were EMG assigned 3.5% (89) and 57.9% (1448), respectively, to these nodes, while META-pipe assigned 5.3% (320) and 49.3% (2913). For the “Moose” dataset EMG classified 15630 sequences (62%), while META-pipe classified 41130 (94%). As in the “Muddy” dataset, trends are similar when ignoring unassigned and eukaryotic fractions, with most identified taxa showing only marginal differences between the two pipelines. The discrepancy observed between the EMG and META-pipe in prediction of rRNA sequences and taxonomic classification relies heavily on the methods, parameters and settings, and the underlying databases used, meaning a more thorough benchmarking of the different methods and databases are needed to determine the sensitivity, specificity and accuracy.

Although we observe comparable results from the taxonomic classification, there is a need for benchmarking of tools for rRNA prediction and classification in addition to dedicated rRNA databases for the marine domain.

### Functional analysis

To gain more insight into the effect of assembling compared to merging paired-end sequencing reads before CDSs prediction and functional assignment, we compared the output results from the functional analysis of the “Muddy” sample (ERS624612) from META-pipe and EMG.

In short, we expect the difference will manifest in three different ways when comparing outputs from the two pipelines. Firstly, longer or full length predicted CDSs would give rise to better functional assignment than shorter CDSs. Secondly, since an assembly will reduce the relative coverage to a consensus sequence (contig), the results will not be quantifiable in the same way as an analysis performed on single or merged reads. Thirdly, assembly will reduce the number of candidate CDSs to a subset containing CDSs from the most abundant organisms in the dataset, depending on the complexity of the dataset, sequencing technology and assembly quality.

**Figure 4.**
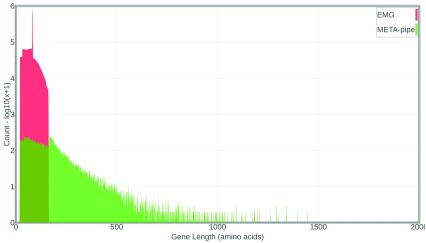
Predicted gene length distribution from META-pipe and EMG pipelines. EMG, EBI Metagenomics Portal.

EMG predict 11 572 617 CDSs (from 12 103 194 merged reads), while META-pipe predicts a total of 47 434 CDSs (from 25 581 assembled contigs > 500 bp), which accounts for 0.4% compared to EMG. We explored the distribution of predicted gene lengths from both pipelines ( Figure 4). On average, META-pipe predicts genes of 155 amino acids in length and the longest gene is 1996 amino acids, while EMG predicts genes of 73 amino acids in length and the longest gene is 162 amino acids.

EMG predicts approximately 1.0 CDS per merged read, while META-pipe predicts 1.9 CDSs on average per contig. Not surprisingly, the longer the contigs the more CDSs predicted, but what effect does this have on the functional assignment of each CDSs and the microbial community as a whole? To answer this question we compared the accumulated number of GO-slim annotations for each analysis using the “Muddy” dataset. In general, the more GO-slim annotations for each CDS, the better description of the molecular function, biological process, and cellular component of gene products will be obtained.

EMG provided a total of 28 942 422 accumulated GO-slim annotations for the predicted CDSs, while META-pipe only provided 565 125 accumulated annotations, which accounts for 0.2% compared to EMG. However, META-pipe provided on average 11.9 GO-slim annotations per CDS, while the number for EMG is 2.5 GO-slim annotations per CDS, which indicated that the longer CDSs predicted a better functional description. Additionally, META-pipe utilizes all available databases shipped with Interproscan5 contrary to the reduced set utilized by EMG, which naturally provides more potential GO annotations per annotated gene. How does this effect the functional assignment of the community as a whole?

**Figure 5.**
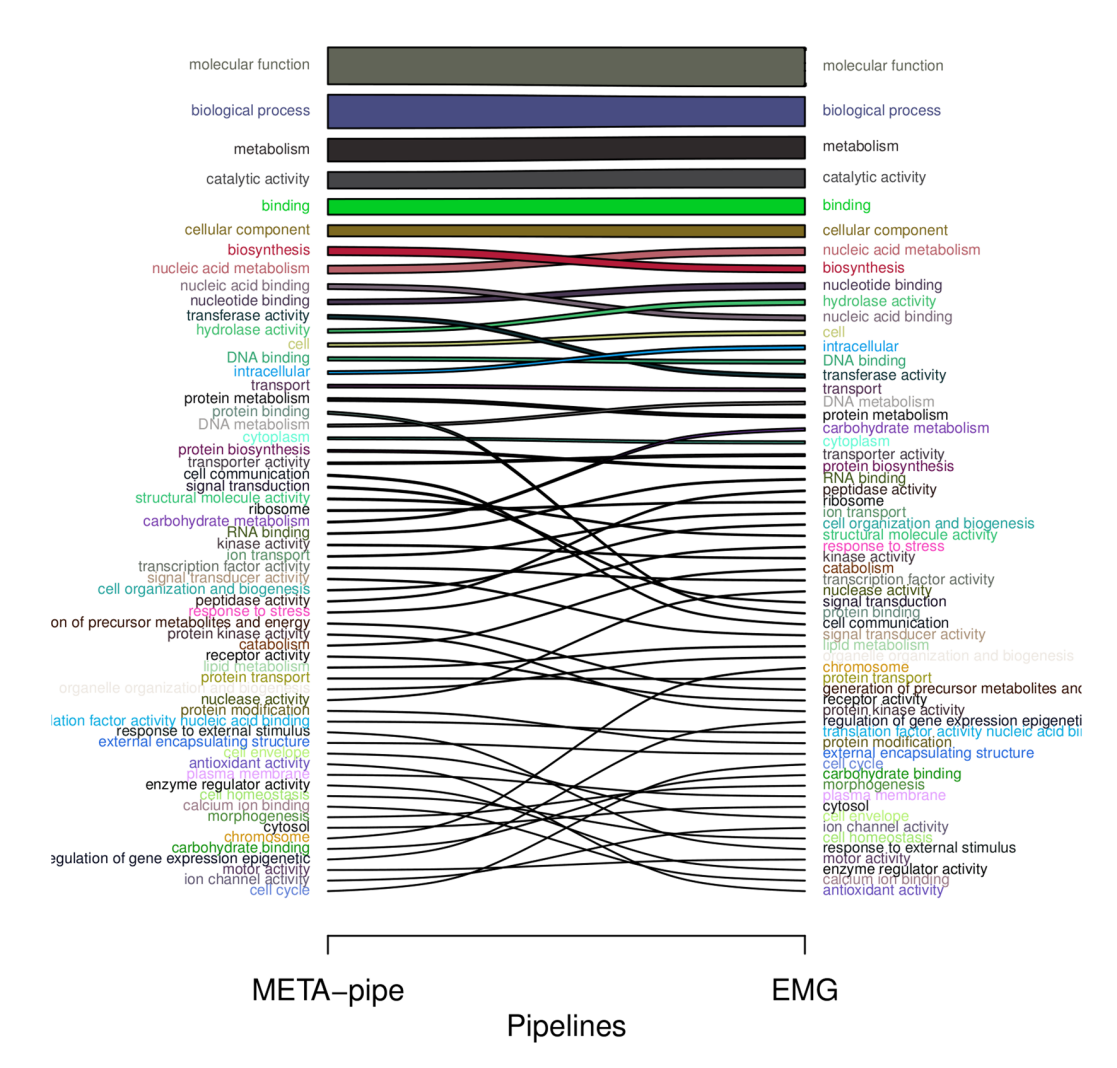
Comparison of counted GO-slim annotations from META-pipe and the EMG pipeline. Thickness of bars corresponds to fraction size of accumulated GO-slim annotations for each pipeline. GO, Gene Ontology; EMG, EBI Metagenomics Portal.

As shown in Figure 5, the effects are relatively small on top-level terms when GO-slim annotations are sorted. Most of the top-level terms (e.g. molecular function, biological process and cellular component) in the GO hierarchy ranks similarly due to accumulative counting (accumulated from counts in lower connected nodes in the diacyclic GO-slim graph). Less common terms are ranked somewhat differently e.g. protein binding, cell communication and carbohydrate metabolism. These differences arise from the observed differences in GO-slim annotation for each predicted CDS in the two pipelines. As META-pipe performs assembly, DNA sequences from low abundance organisms will effectively get excluded from the functional analysis due to insufficient coverage, which in turn changes to GO-profile compared to the EMG-analysis.

The functional assignment of the “Muddy” sample is comparable between EMG and META-pipe. However, a more thorough analysis has to be performed understand the differences observed in low-level GO-terms.

### Harmonization and interoperability

Throughout the project, several changes and improvements were implemented to harmonize, shorten the process time and enrich the output of the two pipelines. In short, masking of homologous sequences before assembly to reduce misassemblies, new databases to enhance functional annotation, optimization and modifications of databases to reduce wall-time. We identified several key steps and file formats within the respective workflows of each pipeline where intermediate data could be interchanged, allowing for potential interoperability between pipelines. Both pipelines have seen improvements since the start of this project. The EMG pipeline is now in version 3.0 ^29^, while a new version/redesign of Meta-pipe is currently in development to improve computational constrains and functionality.

### Gap analysis

In order to develop sustainable ELIXIR services for marine metagenomics, we performed a gap analysis and concluded on four areas where actions are urgently needed. These include the need to: i) standardise metagenomics data generation; ii) establish marine metagenomics resources; iii) develop gold standard pipelines for metagenomics analysis; and iv) explore HPC and storage technologies. A short description on the four recommendations follows.

***Metagenomics data standards***. The context in which marine metagenomics projects are conducted often gets lost, since these data are rarely submitted along with the sequence data. If these contextual data are missing, key opportunities for comparison and analysis across studies and environments are hampered or even impossible to conduct. A metagenomics study should report on each processing step, from contextual data of sampling, through experimental variables of sequencing and metadata of sequence analysis to parameters associated with archiving of the analysed data. Over the past five years standards for describing how a sample was captured and sequenced e.g. for sampling and environment packages, these standards need to be extended to include the whole metagenomics experimental workflow from sample gathering to computational results. As illustrated above, analysis pipelines produce different results on the same input, and comparing the results and understanding whether the differences are real, i.e. coming from the biology of the system under investigation, or whether they are artefacts of the analysis methods, is non-trivial to disentangle.

***Marine metagenomics data resources***. Marine metagenomics research and innovation is limited by the lack of dedicated reference data resources. As indicated by the use of GreenGenes in EMG, existing reference databases are generalized or biased and the contextual data for the records is often incomplete or lacking. Due to the lack of coverage of marine organisms in existing databases, only about one quarter of sequences can be annotated from typical marine samples. To improve the characterization of marine environmental samples, establishment of dedicated data resources for the marine microbial domain is highly needed.

***Gold standards pipelines***. As with most emerging bioinformatics fields, a myriad of tools that perform different types of metagenomics analysis are constantly being published or updated. Pipelines that aggregate such tools are therefore under constant flux. Knowing which tool is the most appropriate to use for specific tasks can be difficult to assess, particular for new researchers entering the field. There is a need to evaluate several types of analysis tool (e.g. preprocessing of reads, prediction of CDSs and taxonomic assignment), and defining gold standard tools and databases.

***High-performance computer and storage technologies***. Marine genomic datasets vary in size, ranging from tens of gigabytes for the typical datasets, to terabytes for projects such as Tara Ocean ^30^, OSD ^31^ and Malaspina (Information available at http://scientific.expedicionmalaspina.es/). Although some pipelines, such as EMG and META-pipe, have been designed for parallel execution on high-performance computer (HPC) clusters, there is a need for exploring more elastic storage and computation resource allocation, e.g. on academic or commercial clouds.

## Conclusion

While there are differences in the respective approaches, EMG and META-pipe provide comparable results. They have their own strengths and weaknesses, and it is clear that the optimal solution for the community would be harmonization and interoperability between the analysis platforms. There is still a need for improvements, e.g. harmonization of the preprocessing step, and improvement of eukaryote taxonomic classification by implementing reference databases for internal transcribed spacers (ITS) and/or large subunit (LSU) rRNAs.

The outcome of the gap analysis has been disseminated to the ELIXIR-EXCELERATE Marine metagenomic infrastructure use case ( https://www.elixir-europe.org/excelerate/marine), which will help to define the requirements and specifications for the establishment of a sustainable ELIXIR marine metagenomics infrastructure.

## Data and software availability

**EBI Metagenomics Portal (EMG):** https://www.ebi.ac.uk/metagenomics/

**META-pipe:** https://galaxy-uit.bioinfo.no (Needs academic user affiliation (FEIDE) or NeLS user login)

The metagenomic sequence reads are available from the European Nucleotide Archive ( http://www.ebi.ac.uk/ena) under the sample accession numbers ERS624612 (muddy), ERS624613 (sandy), ERS624611 (moose) and ERS738393 (sea urchin).

## Supplementary materials

**Supplementary Table 1: Datasets used in the present analysis of the two pipelines.**

.

**Supplementary Table 2: Genomes and Genbank accession numbers included in the in-house marine reference database MarRef.**

.

**Supplementary Table 3: References identified using rRNA in MetaQUAST.**
