# Supplementary material for: ELIXIR pilot action: Marine metagenomics – towards a domain specific set of sustainable services
Source: F1000Res. 2017 Jan 23;6:ELIXIR-70. [Version 1] doi: 10.12688/f1000research.10443.1 (PMC5461914; doi:10.12688/f1000research.10443.1)
Supplement: Supplementary file 1 [file f1000research-6-11253-s0000.tgz › c6a9ebfb-4420-4161-97fb-af95178a97c5.docx]

| Title | Origin | Study accession* | Sample accession* | Sequencing technology | #Reads | Size (bp) |
| --- | --- | --- | --- | --- | --- | --- |
| Muddy | Marine environment | ERP008945 | **ERS624612** | Illumina MiSeq | 18 831 892 | 4,3 billion |
| Sandy | Marine environment | ERP008945 | ERS624613 | Illumina MiSeq | 7 271 336 | 1,8 billion |
| Moose | Gut metagenome | ERP010530 | ERS624611 | Illumina MiSeq | 30 966 692 | 6,3 billion |
| Sea Urchin | Gut metagenome | ERP010530 | ERS738393 | Illumina Miseq | 38 716 356 | 6,6 billion |

**Supplementary Table 1: Datasets used in the present analysis of the two pipelines.**

* Datasets are available at the European Nucleotide Archive
