# Supplementary material for: ELIXIR pilot action: Marine metagenomics – towards a domain specific set of sustainable services
Source: F1000Res. 2017 Jan 23;6:ELIXIR-70. [Version 1] doi: 10.12688/f1000research.10443.1 (PMC5461914; doi:10.12688/f1000research.10443.1)
Supplement: Supplementary file 3 [file f1000research-6-11253-s0002.tgz › 2ec7afcf-4c4e-476a-923d-a579909f4cd3.docx]

**Supplementary Table 2: References identified using rRNA in MetaQUAST.**

| **MetaQUAST with Faeces Datasets** | **MetaQUAST with Marine Datasets** |
| --- | --- |
| Aceivibrio_cellulolyicu_CD2 | Albidierx_errireducen_T118 |
| Adlercreuzi_equolicien | Burkholderi_cenocepci_J2315 |
| Akkermni_muciniphil_ATCC_BAA-835 | Burkholderi_cepci |
| Aneroipe_ccce_DSM_14662 | Cndidu_Niroorcheum_koreeni_MY1 |
| Aneroruncu_colihomini_DSM_17241 | Chondromyce_crocu |
| Bceriovorx_mrinu_SJ | Cireicell_p_357 |
| Bceroide_coprocol | Colwelli_piezophil |
| Bceroide_milieni | del_proeobcerium_PSCGC_5451 |
| Bceroide_plebeiu | Deulibcillum_lkenivorn_AK-01 |
| Buyricicoccu_pullicecorum_12 | Deulobcc_ceoxidn_DSM_11109 |
| Buyrivibrio_croou | Deulobulbu_medierrneu |
| Chlorolexi_bcerium_orl_xon_439_iole_Chl1-2 | Deuluromon_ceoxidn_DSM_684 |
| Cloridicee_bcerium_JC13 | Echerichi_coli_APEC_O78 |
| Cloridium_lepum | Eudore_driic_DSM_19308 |
| Cloridium_mehylpenoum_DSM_5476 | Flvobcerium_rigori |
| Cloridium_ppyroolven | Flvobcerium_gelidilcu |
| Cloridium_phyoermenn_ISDg | Fodinicurv_edimini_DSM_21159 |
| Cloridium_p_01 | Gmmproeobceri_bcerium_SCGC_AAA003-E02 |
| Cloridium_rminiolven_JCM_21531 | Gillii_mrin |
| Cloridium_hermocellum_ATCC_27405 | Glciecol_p_4H-3-75 |
| Colwelli_pychreryhre_34H | Grmell_oreii_KT0803 |
| Coprococcu_come_ATCC_27758 | Ignvibcerium_lbum |
| Cyophg_ermenn_DSM_9555 | Ilumobcer_coccineu_YM16-304 |
| Deulole_pychrophil_LSv54 | Lcinurix_p_5H-3-7-4 |
| Dore_ormicigenern_ATCC_27755 | Leioni_rubr |
| Dore_p_AP6 | Mribcer_p_HTCC2170 |
| Echerichi_coli_IAI39 | Niropin_grcili_3211 |
| Firmicue_bcerium_CAG:822 | Prvulrcul_bermudeni_HTCC2503 |
| Flvobceri_bcerium_BAL38 | Pelobcer_crbinolicu_DSM_2380 |
| Formo_lge | Pelobcer_eleniigene |
| Geobcer_bemidjieni_Bem | Peudoleromon_lnic_T6c |
| Glciecol_rcic_BS20135 | Pychrilyobcer_lnicu_DSM_19335 |
| Keogulonicigenium_vulgre_WSH-001 | Pychromon_rcic_DSM_14288 |
| Lchnopircee_bcerium_6_1_37FAA | Pychromon_ingrhmii_37 |
| Lewinell_peric_DSM_23188 | Synrophobcer_umroxidn_MPOB |
| Mrile_myrionece_DSM_19524 | Thiolklivibrio_ulidophilu_HL-EbGr7 |
| Microcill_mrin | Thiolklivibrio_hiocynodeniriicn_ARhD_1 |
| Moriell_p_PE36 |  |
| Ocdecbcer_nrcicu_307 |  |
| Oleipir_nrcic_RB-8 |  |
| Ocillibcer_p_1-3 |  |
| Prbceroide_merde |  |
| Prprevoell_clr |  |
| Polribcer_dokdoneni |  |
| Pychromon_rcic_DSM_14288 |  |
| Pychromon_ingrhmii_37 |  |
| Pychromon_p_CNPT3 |  |
| Roeburi_homini_A2-183 |  |
| Roeivirg_ehrenbergii |  |
| Ruminococccee_bcerium_D16 |  |
| Ruminococcu_bromii |  |
| Ruminococcu_cllidu_ATCC_27760 |  |
| Ruminococcu_chmpnelleni_18P13 |  |
| Ruminococcu_lvecien |  |
| Ruminococcu_lcri_ATCC_29176 |  |
| Scchrophgu_degrdn_2-40 |  |
| Shewnell_rigidimrin_NCIMB_400 |  |
| Shewnell_pelen_ATCC_700345 |  |
| Shewnell_edimini_HAW-EB3 |  |
| Srepococcu_glcie_SS1219 |  |
| Suliobcer_p_EE-36 |  |
| Suliobcer_p_NAS-141 |  |
| Treponem_cchrophilum_DSM_2985 |  |
| Vibrio_plendidu |  |
| Vibrio_plendidu_LGP32 |  |
