# Supplementary material for: ELIXIR pilot action: Marine metagenomics – towards a domain specific set of sustainable services
Source: F1000Res. 2017 Jan 23;6:ELIXIR-70. [Version 1] doi: 10.12688/f1000research.10443.1 (PMC5461914; doi:10.12688/f1000research.10443.1)
Supplement: Peer review history

# Peer review: Referee response for version 1

**DOI:** 10.5256/f1000research.11253.r19596

**Published:** 2017-05-31

**Authors:** Anders Blomberg

**Affiliations:** 1 Department of Marine Sciences, Lundberg Laboratory, University of Gothenburg , Gothenburg, Sweden

Metagenomics has a great potential to influence our understanding of the complex ecology of biotopes, including marine waters. Despite the impressive speed of generating sequence data, the analyses pipelines are not as well developed and standardized. This article describes a comparison between two analysis pipelines and how they perform on different types of sequence data. The main methodological difference between the two pipe-lines tested is if the anaysis is done on the read-level (EMG) or at the contig-level (META-pipe). This will of course have a major influence on the results obtained, which is in essence what this study aims to outline. The manuscript also has a link to a nice webinar that explains parts of the background, technical details, challenges and some of the results.

 Major critique/comments:

1. Why a specific marine metagenomics pipeline? Why could not this service be generic - independent on where the organisms live (marine, soil, stomach, flowers, etc....).
  This issue is addressed in the webinar, but not in the paper, e.g. marine samples/sequences are taxonomically complex and with really high genetic/sequence diversity. There might be more reasons. These reasons for a specific marine pipeline should be outlined in 1-2 sentences in the paper.
2. Why picking unpublished data for the test? Anything specifically general with this data? Or could there be very specific biases and technical problems with this data? This should be outlined and described. They should also consider using some already published data for their comparison. 
  In addition, the data analysed is based on comparably long-read Illumina reads - 250nt and 300nt. Plenty of metagenomics data has been, and will be, collected using more standard length reads (≈ 125nt). Please discuss, e.g. in the conclusion part, to what extent this selection of example data could have had an impact on the obtained results. 
3. In the Conclusion section they state: " While there are differences in the respective approaches, EMG and META-pipe provide comparable results. "
  But do they really show similar results? There appear to exist huge differences between the two programs that are also highlighted earlier in the text:
  p.6, rc, " While META-pipe was able to predict 6584 16S rRNA sequences, EMG predicted 4339 in the “Muddy” dataset (Figure 2)."
  p.6, rc, " In the “Muddy” dataset, EMG classified 2500 sequences (58%), while META-pipe was able to classify 6119 (93%)."
  p.8, lc, " EMG predict 11 572 617 CDSs (from 12 103 194 merged reads), while META-pipe predicts a total of 47 434 CDSs (from 25 581 assembled contigs > 500 bp), which accounts for 0.4% compared to EMG.
  p.8, rc, " EMG provided a total of 28 942 422 accumulated GO-slim annotations for the predicted CDSs, while META-pipe only provided 565 125 accumulated annotations, which accounts for 0.2% compared to EMG."
  I think there statement about "comparable results" should be modified and differences also highlighted in the Conclusion.
4. Finally, should one recommend that both pipelines are used in analyses before publication, and that results are being reported? And if so, what about other pipelines? How do they see that this challenge (which is a great problem in the comparison of results between studies using different analysis pipelines) should be handled in the future?

 

 Minor comments:

 Abstract

 In the last sentence of the abstract it says:

 "In this paper, we summarize some of the results from the ELIXIR pilot action “Marine metagenomics – towards user centric services”. Shouldn't this be the same as in the title?

 Page 3, left column (lc), line 7

 I am not sure I see why replication would be hard given the information in publications - databases are not per see a guarantee for higher transparancy in information handling. Even if the access to the data might be easier. The statement should be modified. Or do they mean "results" and not "analyses" are hard to replicate?

 p.3, lc, l.15

 Please provide a short overview of the types of metagenomics pipeline that are available at this stage. Please explain to the reader why EMG and META-pipe were selected for comparison? Anything that make this comparison particularly valid?

 p.4, lc, l.3

 preform - > perform

 p.4, rc, l.18

 Give arguments for why Kmer = 31 was selected. Are there reasons to believe the results would have been different if another Kmer had been used?

 p.4, right column (rc), l.4 from bottom

 Please explain "biomes".

 p.6, lc, l.33

 They state that META-pipe is reluctant to classify on species level. Can one explain to the reader why that is?

 p.6, lc, l.35

 Be more specific - how was "better" defined?

 Table 2

 Just to be sure - do they mean prokaryotic + eukaryotic?

 Table 2

 Why can't EMG do eukaryotes?

 Figure 4

 What do we really learn from this figure?

 Figure 5

 How have the annotations been sorted?

 p.9, lc, l.3

 to - > the

 p.10, lc, l.10 from bottom

 They talk about gold standard tools. But these might differ dependent on the data - technical problems; community complexity; lengths of reads; .... Can they be a bit more specific for how they see that this "golden standard" can be reached?

I have read this submission. I believe that I have an appropriate level of expertise to confirm that it is of an acceptable scientific standard, however I have significant reservations, as outlined above.

# Peer review: Referee response for version 1

**DOI:** 10.5256/f1000research.11253.r21907

**Published:** 2017-04-18

**Authors:** Takashi Gojobori

**Affiliations:** 1 Computational Bioscience Research Center (CBRC), Biological and Environmental Sciences and Engineering Division, King Abdullah University of Science and Technology (KAUST), Thuwal, Saudi Arabia

Title and Abstract

 The title can be more precise in reflecting the contents. For example, “ELIXIR pilot action: Comparisons of two representative pipelines of metagenomics between EMG and META-pipe”.

 In Abstract, the outcome of the two pipeline comparisons may be mentioned.

Article Content

 The design, methods and analysis of the results from the study been explained well and they are appropriate for the topic being studied. However, I have the following comments:

1. In the studies of metagenomics, there are essentially two ways of picking up genomic fragments from the DNA samples: (a) Amplicon-oriented approach and (b) Random shotgun approach.
  For approach (a), rRNAs can be targets for sequencing whereas for approach (b) any genomic fragments can be targets. Naturally, approach (a) can be used for phylogenetic identification only, while approach (b) can be used for not only phylogenetic identification but also functional analysis.
  If the authors explain about these two approaches clearly in the text, the whole context can be easier to understand to the readers who do not have the expertise of metagenomics.
2. Marine metagenomics is one of metagenomic studies. The two pipelines explained in the present paper are basically for metagenomics in general, but not specialized for “marine” metagenomics. Then, the authors may be requested to clarify which points in the pipelines or in the methodologies are keen differences between metagenomics in general and “marine” metagenomics.
3. Although the main part of the present paper is on differences between the two pipelines. In particular, whether the genomic fragment assembly is conducted or not appears to lead to huge differences of the outcome between the two pipelines. This is very important notion. According to our experiences of marine metagenomics, we have already recognized that the fragments assembly produces more identifiable OTUs and function in the annotation process. Therefore, we usually conduct the fragment assembly.
  However, the authors did not mention anything about it in Conclusion (Also see below).

Conclusion

 In Conclusion, the authors have stated that while there are differences in the respective approaches, EMG and META-pipe provide comparable results. I do not think so. As mentioned above, the author should state the main different point between two pipelines, because they are not really “comparable”.

Data

 I think that enough information has been provided to be able to replicate the experiment.I also think that the data are in a usable format/structure and all the data have been provided.

I have read this submission. I believe that I have an appropriate level of expertise to confirm that it is of an acceptable scientific standard.

# Peer review: Referee response for version 1

**DOI:** 10.5256/f1000research.11253.r19670

**Published:** 2017-02-13

**Authors:** Marla I. Trindade, Leonardo van Zyl

**Affiliations:** 1 Institute for Microbial Biotechnology and Metagenomics (IMBM), Department of Biotechnology, University of the Western Cape, Bellville, South Africa; 2 Department of Biotechnology, University of the Western Cape, Bellville, South Africa

The authors, using 4 different metagenome datasets, compare the assembly and annotation results of 2 pipelines (META-pipe and EMG). They additionally compare the assemblies after also filtering out rRNA reads.

 As presented by the authors, the discrepancy observed between the 2 pipelines, particularly in predicting the taxonomic classification, was significant, and alarmingly different. While such a limitation is acknowledged by many published studies, and accepted in practice, I 100% agree with the authors that it is crucial for rigorous benchmarking to be conducted, and they rightly conclude that action is urgently needed - it goes against all research principles to present conclusions and hypotheses when results are generated using tools and methods which are accepted to completely bias the outcome. What actual scientific value do such studies offer given the discrepancies? Thus, for this reason, the study by Robertsen et al should be indexed so as to raise further awareness to the danger of not paying attention to the proper curation of next gen sequence data generation and analysis.

 I do however have some major and minor questions / corrections which need to be addressed before this study can be approved:

**Major:**

1. The authors provide absolutely no information of how the samples were collected and how the DNA was prepared. Given that the authors in their gap analysis, themselves recognise that this kind of detail should be reported in every metagenomic study, it is ironic that they do not do so even if the purpose of the manuscript was not to describe the content of these metagenomes. Furthermore, the Gap analysis should also address the biases that the mDNA extraction and sequencing technology introduce, which are also well documented. 
2. Please confirm whether the figures presented for the "aligned to reference" in Table 1 represents the combined results when using the MarRef and the de novo generated database? It is confusing because in the methods section it is stated that the sediment samples were analysed using the MarRef whereas the others were done using the MetaQUAST-generated database; however, in the results section the marine samples are reported to have been analysed using both databases. Also, what were the cut-off values used for the reference alignments, and were parameters modified to try and increase the %assemblies, or do these represent the best possible outcome?  
3. Irrespective of the answer in the above, the % that could be referenced was very low (the highest was 0.262%), representing a minute proportion of the sequence data generated. If I understood correctly, the #misassemblies refers to only the % of sequences that could be referenced (ie. a minute proportion of the sequence data), and thus I do not think this is a very informative factor with which to compare the different assembly procedures. i.e. if misassembly is only judged on between 0.001% and 0.284% of the dataset this might not be an accurate reflection of assembly issues, as suggested by the authors.
4. The choice of assembler and assembly parameters could have a huge impact on the outcome on the META-pipe pipeline. Have the authors, and pipeline administrators, satisfied themselves that MIRA is the best assembler (SPADES, IDBA-UD, collaboration with CLC Genomics?). The fact that when you had longer contigs (faecal dataset), the number of missasemblies increased significantly, points to assembly issues. See a very recent study be Hesse et al 2017 (with relevant references within) which specifically addresses these issues.
5. The authors use a non-validated dataset to determine the best pipeline (or at least differences). It would’ve been preferable to use a curated dataset of known composition, to know exactly what the ideal outcome should’ve been. A good example of this is the taxonomic assignments of the two pipelines. The authors finish paragraph 7 on page 6 with “more thorough benchmarking of the different methods and databases are needed to determine the sensitivity, specificity and accuracy”. Had they used a curated dataset, they would know which pipeline gives a more accurate picture of the taxonomic composition of the uploaded dataset. The study suffers the same issue when looking at functional classification.
6. I agree that dedicated effort and resources needs to be established to ensure increased population of the sequence databases with marine derived genome data. Please can the authors clarify whether they are proposing there to be dedicated marine databases - if this is the case I do not support this notion. From an ecological perspective it would be interesting to make connections with terrestrial systems, if they exist. Only a comprehensive database could help you establish these links. If purely for bioprospecting, perhaps this is less of an issue. Or did the authors simply mean that increased research effort / focus is needed?
7. In their gap analysis the authors propose the need for evaluating analysis tools and defining gold standard tools and databases. Standardization of metagenomic data generation is a great idea, similar to the MIQE guidelines for real-time PCR, but very difficult to implement in practice. The pipelines should always allow some flexibility to accommodate datasets outside the “ideal”. Can they propose who should take on this responsibility, or how to coordinate such a task?
8. No comparison was made to MG-RAST (278,783 metagenomes), one of the most widely used metagenomic analysis pipelines. If a “gold standard” pipeline is to be created, surely it should also be done in consultation with all large groups involved with such analyses.
9. If I understand Figure 5 correctly, the more parallel lines we have the more similar the predictions of the two pipelines? Can the authors provide some quantitative value to summarize the information given in the Figure? Otherwise the display of the results of this analysis seems a bit arbitrary as my only other two references are a figure with all parallel lines (1), and one where there are no parallel lines (0). I acknowledge this may not be easily doable.

**Minor:**

1. Pg4, line 13: “OUT” change to “OTU”
2. Pg5, end of the first paragraph: "and results was visualized" should be changed to "and results **were** visualized".
3. Pg5, last paragraph:  Figure 4 does not really add value. What it displays is fully expected and will only change for the META-pipe pipeline depending on the environment being sequenced as well as “depending on the complexity of the dataset, sequencing technology and assembly quality”.
4. Pg8, 3rd paragraph: "the longest gene is 1996 amino acids". Although it is understood what the authors mean, it is incorrect terminology and this needs to be corrected in this paragraph and also in the Figure 5 legend. A gene is denoted in base pairs, and protein in amino acids.
5. Pg9, paragraph 2: "analysis has to be performed understand" needs to be changed to "analysis has to be performed **to** understand".
6. Pg10, 3rd paragraph: "difficult to assess, particular for new researchers" should be changed to "difficult to assess, particular **ly** for new researchers".

We have read this submission. We believe that we have an appropriate level of expertise to confirm that it is of an acceptable scientific standard, however we have significant reservations, as outlined above.
